# Participants’ Engagement With Telephone Support Interventions to Promote Healthy Feeding Practices and Obesity-Protective Behaviours for Infant Obesity Prevention

**DOI:** 10.3389/fendo.2022.868944

**Published:** 2022-05-02

**Authors:** Mahalakshmi Ekambareshwar, Huilan Xu, Chris Rissel, Louise Baur, Sarah Taki, Seema Mihrshahi, Li Ming Wen

**Affiliations:** ^1^The Centre of Research Excellence in the Early Prevention of Obesity in Childhood, Sydney School of Public Health, The University of Sydney, Camperdown, NSW, Australia; ^2^Sydney School of Public Health, Faculty of Medicine and Health, The University of Sydney, Camperdown, NSW, Australia; ^3^Health Promotion Unit, Population Health Research and Evaluation Hub, Sydney Local Health District, Camperdown, NSW, Australia; ^4^Discipline of Child and Adolescent Health, The University of Sydney, Camperdown, NSW, Australia; ^5^Weight Management Services, The Children’s Hospital at Westmead, Westmead, NSW, Australia; ^6^Department of Health Systems and Populations, Faculty of Medicine, Health and Human Sciences, Macquarie University, Macquarie Park, NSW, Australia

**Keywords:** mobile telephone interventions, engagement, mHealth, childhood obesity prevention, process evaluation

## Abstract

**Background:**

Participant engagement with program interventions is vital to support intended behaviour changes and outcomes. The aim of this research was to investigate participant engagement with the Communicating Healthy Beginnings Advice by Telephone (CHAT) program, an early childhood obesity prevention program that included interventions for promoting healthy infant feeding practices and obesity-protective behaviours *via* telephone, and whether engagement with the telephone support program varied by participants’ sociodemographic characteristics.

**Methods:**

This study used de-identified CHAT program data of participants who received the interventions *via* telephone. Data analysed included 1) participant engagement in telephone support from late pregnancy to 12 months of child's age, 2) demographic characteristics collected at late pregnancy and 3) intervention providers’ observations and notes (qualitative data) for 10 participants from each engagement group (low, medium, high) to explore issues discussed during telephone support.

**Results:**

Call completion rate by participants was above sixty percent for all six stages of the telephone support program with more than half of the participants (57%) demonstrating high level of engagement. We found that participants’ country of birth, employment status and annual household income were predictors of engagement with the telephone support provided in the CHAT program. The odds of participants’ engagement with the telephone support program were 1.68 times higher for Australian born (95% CI 1.07 – 2.62), 1.63 times higher for participants who were employed (95% CI 1.01 – 2.66) and 1.63 times higher for participants with annual household income ≥AUD$80,000 (95% CI 1.02 – 2.60).

**Conclusions:**

Participant engagement with the program interventions was good. Participants’ engagement with the telephone support program was significantly associated with certain socio-demographic characteristics. Australian born participants, and participants associated with higher household income and employment engaged significantly more with the telephone support provided in the CHAT program. Additionally, the program engaged more participants older than 30 years of age and those who spoke English at home. The program provided unintended personal benefits to some participants with high engagement level due to their various psychosocial needs such as domestic violence, mental health and sleep related issues. Although not an intended benefit of the intervention, psychosocial needs of participants were met which was a likely factor for mothers’ engagement with the program. This is an important factor that needs to be considered while implementing future programs or scale up of this program.

## 1 Introduction

Obesity is recognised as a global health problem and prevention of obesity is a public health priority. In general, prevention research has received increased attention. For over a decade, in the case of early childhood obesity prevention interventions, the focus has been to commence early from when women are pregnant (1, 2). However, recruitment of participants to a program and keeping mothers and caregivers of infants engaged at a busy time of their lives remains a challenge (3–5).

Although face-to-face delivery of child obesity prevention interventions has been widely accepted by participants, some barriers have been identified that may inhibit uptake and attendance. Barriers include lack of transportation and travel time (6); poor enrolment and attendance (7); feelings of stigmatisation, fear and guilt (4, 7); and fear of being judged in face-to-face sessions for not possessing the desired skills (8, 9). To overcome the challenge of requiring participants’ presence at designated locations for in-person participation, and to make it less onerous on participants, more recently, interventions have been delivered remotely *via* telephone calls or text messages (1, 2). Intervention delivery *via* telephone calls provides participants with an opportunity to receive personalised health advice and to resolve questions about the program content (10, 11). Intervention delivery *via* a combination of telephone calls and text messages has the advantage of reminding participants *via* text messages about the interventions delivered *via* telephone calls (10, 11).

The Communicating Healthy Beginnings Advice by Telephone trial (CHAT program) is a three-arm randomised controlled trial (RCT) that delivered stage-based health behaviour change messages to women *via* nurse-led telephone calls or text messages (2). Intervention delivery commenced from the third trimester of pregnancy to 12 months of child’s age and beyond. In behavioural intervention trials, it is important to evaluate the process that occurred during intervention delivery and the extent to which participants engaged with the interventions (12). This research was one of four studies solely conducted to evaluate the process of delivering the CHAT program. There were other studies that evaluated the clinical outcomes of the CHAT trial (13, 14). In brief, telephone support was effective in promoting the appropriate timing of the introduction of solid foods and early-start tummy time (at 6 months). Both the nurse-led telephone support and SMS interventions were effective in reducing screen time and bottle use at bedtime (at 12 months) (13). At two years of child's age, the telephone or SMS support intervention was effective in increasing ‘no bottle use’ at bedtime, telephone support showed more effects than SMS on reducing screen time and eating behaviours (14). Meticulously describing the actual exposure to the intervention, the engagement experience of those exposed to the intervention (participants) and the intervention itself forms part of process evaluation, all of which are crucial towards understanding the success of interventions (15).

Program engagement is crucial to any intervention delivery for participants to adapt and to practice the intended behaviours (3, 4). Participant engagement is pivotal to participants’ adherence to intended behaviour changes and outcomes. In fact, dose-response is often expressed as dose received, a characteristic of the target audience that assesses the engagement of participants with the intervention (16). Participant engagement has been investigated in community-based childhood obesity prevention or treatment programs (7, 8); in digital/online delivery (17, 18); *via* web where more intense engagement led to better outcomes (19); and *via* mobile phone app where engagement level positively correlated with the combined use of app, email and intervention exposure for a longer period (17, 18). Remote contact with a health professional *via* email, telephone or text messages positively influenced enrolment in program (20).

To our knowledge, engagement of participants in infant obesity prevention interventions delivered *via* telephone calls has not been investigated, and characteristics of those who were engaged remain unexplored. Although telephone calls and text messages were used to deliver interventions in the CHAT program, it is not possible to measure actual engagement with text messages since we cannot know if participants actually received, opened and read the text messages (21). The purpose of this research is to investigate engagement of participants with the telephone support provided in the CHAT program, to inform the development of future programs. We evaluated whether participants’ engagement with the program was associated with their sociodemographic characteristics.

## 2 Materials and Methods

### 2.1 Study Context

The CHAT program was conducted across four Local Health Districts (LHDs) within New South Wales (NSW) in Australia. Three LHDs were located within metropolitan Sydney and one LHD was in regional NSW, where pregnant women were recruited at eight hospital sites between February and July 2017. The study protocol, eligibility criteria, recruitment process and outcomes are reported in detail elsewhere (2, 11, 13, 20). In brief, CHAT is a three-arm RCT that compares: mailed Healthy Beginnings booklets plus telephone support (telephone); to mailed Healthy Beginnings booklets plus text messages; to the control arm. Interventions were stage-based and provided at six time points following key developmental milestones from the antenatal period (third trimester) until the end of first year of the infant’s life. The control arm participants were mailed general infant safety promotion materials. Usual care on infant development and safety is delivered by local child and family health services that are not mandatory.

### 2.2 CHAT Interventions

Briefly, the focus of the stage-based interventions that were delivered to participants *via* telephone calls from recruitment until six months of children’s age were on breastfeeding, introduction of “tummy” time (allowing babies time lying prone on their abdomen while they are awake) within four weeks, introduction of solid food at six months. In addition to the above, between six and twelve months of children’s age, the focus of the intervention was on drinking from a cup, no screen time for children, children’s activity time and not offering food for reward to children.

The key messages of the telephone interventions intended to influence target behaviours were: an increased breastfeeding rate, and duration at 6 months and at 12 months of children’s age; an appropriate timing of introduction of solids at 6 months; the commencement of “tummy time” within 4 weeks of birth and increased rate of practising “tummy time” at 6 months; an increased rate of using cup and drinking water at 12 months; nil or reduced child TV screen time at 12 months of children’s age. The outcomes of the CHAT program, including child BMI z score at 12 months, are reported elsewhere (13).

### 2.3 Data Collection and Measurement

Participants’ socio-demographic characteristics were assessed at baseline. The demographic summary of all CHAT participants is published elsewhere (13). Questions from the NSW Adult Population Health Survey 2003 were used to measure participants’ sociodemographic characteristics (22), which included participants’ age, country of birth, language spoken at home, education level, annual household income, employment and marital status, and parity of participants. Participants’ demographic and socioeconomic information were categorised into groups based on their engagement level.

#### 2.3.1 Participants’ Engagement With Telephone Support Intervention

We measured participants’ engagement as the number of completed telephone support calls recorded on the project implementation database using Research Electronic Data Capture (REDCap) (23). Telephone calls were considered completed (and included in our assessment of engagement) where participants answered the calls, and the intervention components were delivered as intended and recorded by the intervention providers. Where calls were directed to voicemail messages or were interrupted, these were not included in the number of completed calls. We did not include the amount of time spent on calls in our assessment of engagement since in some instances the intervention providers spent time on participants’ contextual/psychosocial issues (11, 24). Four staged telephone calls were delivered to participants until six months of child’s age, and two more between 6 and 12 months of child’s age.

We assessed engagement level of participants with the telephone intervention based on the number of telephone calls answered in the first six stages of the intervention i.e., from antenatal stage to 12 months of child’s age. We categorised engagement into three levels based on the number of calls answered - participants who answered less than three calls (lowest third) were regarded as ‘low engagement’, those who answered three to four calls (middle third) as ‘medium engagement’ and greater than four calls (upper third) as ‘high engagement’.

### 2.4 Data Analysis

#### 2.4.1 Quantitative

Statistical analyses were carried out using Stata 13 (25). All P-values are two sided and statistical significance was set at the 5% level. To examine the associations between participants’ baseline socio-demographic characteristics and their engagement in telephone support, Pearson’s Chi-squared tests were conducted using data from the telephone support group. Number and percentage were reported. Ordinal logistic regression models (Proportional odds model) were built to determine whether participants’ socio-demographic characteristics were associated with their level of engagement in telephone support intervention at 12 months of child's age. Participants’ socio-demographic variables that were significant on bivariate analyses using Pearson’s chi-squared tests with P<0.25 were entered in the ordinal logistic regression models. The least significant variables were progressively dropped until only those with P<0.05 remained. Variables dropped from the model were then entered into the model individually to assess for confounding factors. The Brant test of proportionality of odds showed that parallel regression assumption was not violated and indicated the proportional odds mode was appropriate. Adjusted odds ratio (AOR) with 95% confidence interval (CI) were calculated.

#### 2.4.2 Qualitative

We randomly selected 10 participants from each engagement group (low, medium, high) to further explore intervention providers’ notes on these participants, and to see whether the issues raised and discussed during the telephone calls varied with participants’ engagement levels. Participants’ notes were recorded by intervention providers on REDCap. These data were extracted and transferred to NVivo for analysis (26).

## 3 Results

### 3.1 Quantitative Data

**Table 1** provides the number of staged telephone calls completed by participants in the telephone support program. There were a total of 1630 telephone calls answered by 386 participants in the telephone support group for the first six interventions delivered from the third trimester of pregnancy to 12 months’ of child’s age. An average of four calls were completed by each participant. The completion rate was above 60% for all 6 stages of telephone support, the highest completion (87%) at stage 2 (one month of child’s age) and the lowest completion (61%) at stage 1 (late pregnancy).

**Table 1:** Number of completed telephone calls for each stage of intervention.

Staged telephone call	Number of completed calls (out of 386) n (%)
1 (antenatal)	234 (61)
2 (baby age 1month)	336 (87)
3 (baby age 3 months)	283 (73)
4 (baby age 5 months)	245 (63)
5 (baby age 8 months)	269 (70)
6 (baby age 12 months)	263 (68)

**Figure 1:**
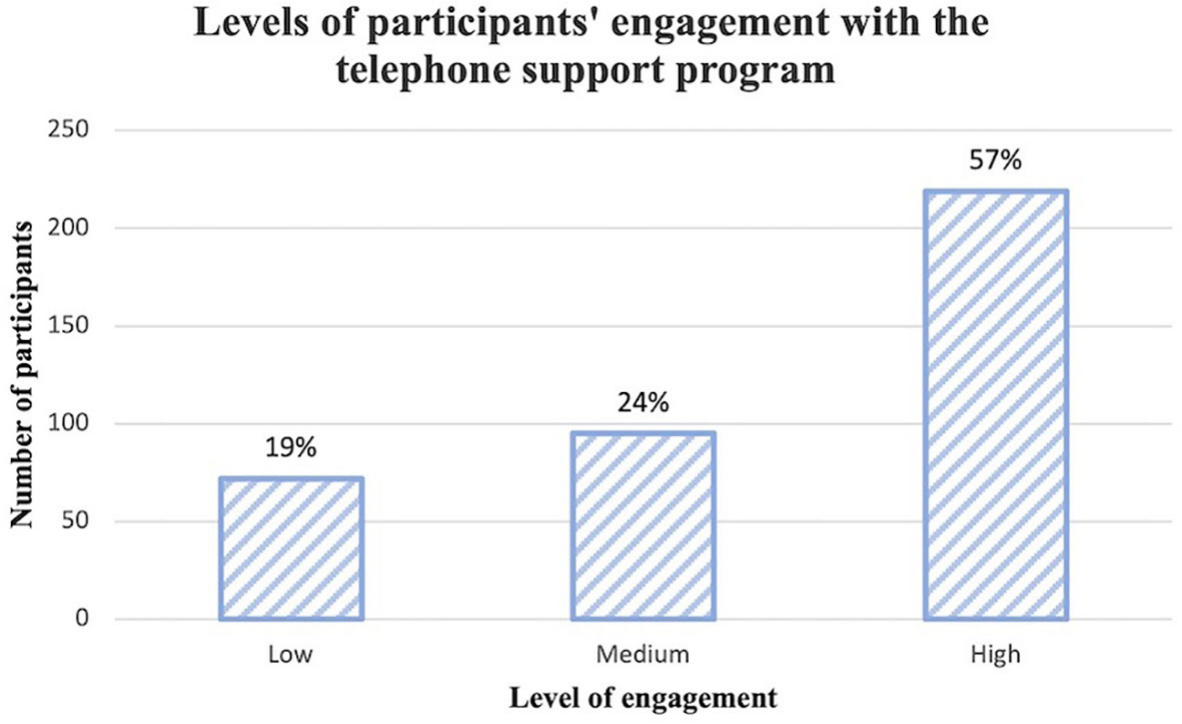
Levels of participants’ engagement with the telephone support program.

**Figure 1** represents the engagement level of participants based on the number of staged calls answered. Of the 386 participants in the telephone support group, 219 women answered more than four of the six calls and demonstrated high level of engagement with the telephone support program. 95 participants had mid-level of engagement and 72 had low level of engagement with the program.

**Table 2** shows that, using bivariate analyses, mothers’ age, country of birth, language spoken at home, educational level, employment status and annual household income were significantly associated with their level of engagement with the telephone support program at 12 months of child's age. Marital status and parity of mother were not significantly associated with their level of engagement with the program.

**Table 2:** Comparisons of the socio-demographic characteristics between those who had high, medium and low levels of engagement.

Mother’s baseline demographics	Engagement			
	High n (%)	Medium n (%)	Low n (%)	P
**Age (years)**				0.003
16-24	13 (39.5)	7 (21)	13 (39.5)	
25-29	50 (54)	18 (20)	24 (26)	
30-34	79 (59)	34 (25)	22 (16)	
35-49	77 (61)	36 (29)	13 (10)	
**Country of birth**				0.023
Australia	94 (66)	27 (19)	22 (15)	
Overseas	125 (51)	68 (28)	50 (21)	
**Language spoken at home**				0.021
English	130 (63)	47 (23)	30 (14)	
Other	89 (50)	48 (27)	42 (23)	
**Education level**				0.035
University &	145 (56)	73 (28)	42 (16)	
Up to HSC/TAFE*	74 (59)	22 (17)	30 (24)	
**Employment status**				0.001
Employed	154 (63)	56 (14)	34 (23)	
Other	65 (46)	39 (27)	38 (27)	
**Annual household income**				0.002
<$ 80,000	62 (48)	36 (28)	31 (24)	
≥$ 80,000	138 (65)	48 (22)	27 (13)	
Don’t know	19 (43)	11 (25)	14 (32)	
**Marital status**				0.556
Married/de-facto partner	203 (57)	87 (25)	63 (18)	
Other	15 (47)	8 (25)	9 (28)	
Unknown	1 (100)	0 (0)	0 (0)	
**First time mother**				0.717
Yes	121 (58)	48 (23)	40 (19)	
No	98 (55)	47 (18)	32 (27)	

^*^HSC, Higher School Certificate (Year 12); ^TAFE, Technical and Further Education.

In a multivariable model the odds of participants’ engagement with the telephone support program were 1.68 times higher for Australian born (95% CI 1.07 – 2.62), 1.63 times higher for participants who were employed (95% CI 1.01 – 2.66) and 1.63 times higher for participants with higher annual household income (95% CI 1.02 – 2.60) (**Table 3**).

**Table 3:** Associations of participant socio-demographic characteristics with engagement in telephone support program using ordinal logistic regression.

Socio-demographic characteristics	AOR* (95% CI)	P
Mothers’ country of birth		0.023
Other	–	
Australia	1.68 (1.07 – 2.62)	
Annual household income		0.041
<$80,000	–	
≥$80,000	1.63 (1.02 – 2.60)	
Mothers’ employment status		0.047
Other	–	
Employed	1.63 (1.01 – 2.66)	

^*^AOR, adjusted odds ratio; variables in the table adjusted each other.

### 3.2 Qualitative Data

**Figure 2:**
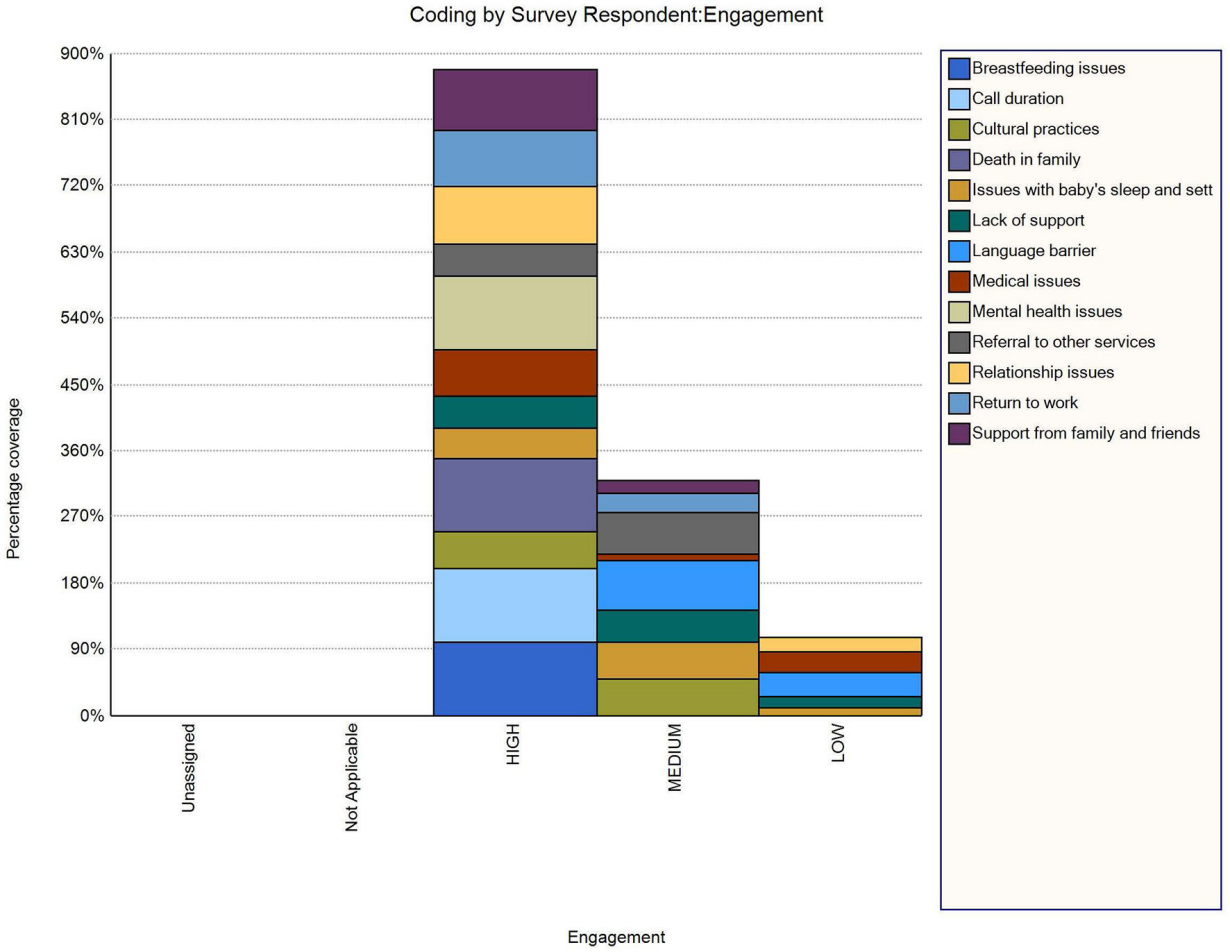
Representation of issues discussed during intervention provision by participants’ engagement level and as recorded by intervention providers who delivered telephone support.

For the sample of 30 participants from the three engagement level groups (low, medium, high), intervention providers’ notes demonstrated that a range of issues, including psychosocial concerns, were discussed at the time of intervention delivery. Concerns that were raised and recorded by the intervention providers included: medical issues (12); mental health issues (1); baby’s sleep and settling (11); return to work (8); referral to other services (8); and lack of support (8) (**Table 4**). **Figure 2** represents concerns that were raised by participants based on the three levels of engagement: participants in the high engagement category discussed various issues outside of the intervention content than those in the lower engagement categories.

**Table 4:** Representation of themes discussed during intervention provision from intervention providers’ notes on a sample of participants (N=30) using NVivo.

Name	References
Breastfeeding issues	1
Call duration	1
Cultural practices	2
Death in family	1
Issues with baby’s sleep and settling	11
Lack of support	8
Language barrier	3
Medical issues	12
Mental health issues	1
Referral to other services	8
Relationship issues	5
Return to work	8
Support from family and friends	6

## 4 Discussion

To our knowledge, engagement of participants in infant obesity prevention interventions delivered *via* telephone calls was not previously investigated, and sociodemographic characteristics of those who engaged were not previously reported. We set out to explore participants’ engagement with infant feeding interventions delivered *via* telephone and whether engagement was related to participants’ sociodemographic characteristics. Overall, participant engagement with the program interventions was good with more than half of the participants (57%) demonstrating high level of engagement by answering more than four of the six calls; and only less than one-fifth of participants (19%) answering less than 3 calls. We found that participants born in Australia, who were employed, and had a higher annual household income were significantly more likely to engage with telephone calls in the CHAT program. To our knowledge, this is the first study to measure the relationship between engagement and participants’ characteristics in a trial that delivered infant feeding practices *via* telephone calls.

Participants in the 30-49 years age group were more likely to engage with the CHAT program. We were not able to locate a similar finding regarding participants’ engagement in the childhood obesity prevention literature. However, the positive association of participant engagement with increasing age is consistent with studies in the general population showing that interventions delivered digitally (27) or *via* eHealth (28) demonstrated increased uptake of and satisfaction with interventions with increasing age.

Employment and household income of participants were positively associated with engagement in the CHAT program, with higher level of engagement by participants with annual income ≥AUD$80,000. Previous studies have attributed higher eHealth service attendance to higher socioeconomic status and educational attainment (28), and families with lower socioeconomic status attended treatment infrequently in an infant obesity management intervention (6). Low socioeconomic status has been a strong predictor of program non-completion in paediatric and youth weight management programs (29–31). However, in a community-based weight management study, families with lower education levels achieved better outcomes with a greater number of sessions (32). Consistent with previous literature participants from higher socio-economic background appeared to seek out ‘reliable’ information (8).

Participants who spoke English at home were more likely to engage with the CHAT program in comparison to those participants who spoke other languages. Previous research has shown that ethnicity (Anglo-ethnic and largely identified by language spoken) was positively associated with access and uptake of eHealth services (28). Engagement and access to mainstream services and interventions have been particularly challenging for participants from other cultural backgrounds who speak a language other than English as their main language (9) and have been associated with inconsistent program attendance (29, 33–35). Consequently, there has been a need for cultural adaptation and translation of interventions to reach broader populations (36).

It is evident from our analyses of the intervention providers’ notes that demographic characteristics were not the only indicators of engagement with the CHAT program. Participants with high engagement level had various psychosocial needs such as addressing domestic violence, mental health, sleep related and other issues. Psychosocial characteristics are of great interest for subsequent studies to predict engagement. During the later stages, mainly participants with health and personal concerns sought help and found value in telephone calls and these calls were quite lengthy (24). Although most calls were under 30 minutes, participant-driven telephone conversations with personal concerns led to longer call duration which in some instances were more than an hour (24). Psychosocial concerns that participants were most concerned about, included medical issues; mental health issues; baby’s sleep and settling; participants’ return to work; referral to other services; and general lack of support available to them. Addressing the psychosocial needs of participants was not the primary focus of the CHAT program but clearly there is a need.

Recruitment of participants to an obesity prevention program and keeping mothers and caregivers of infants engaged at a busy time of their lives remains a challenge that could be overcome by telephone calls. The findings of this study indicate that without addressing the challenges that participants may experience at the time of intervention delivery, it is highly unlikely to engage participants to address factors related to their child’s health such as desirable behaviours around nutrition, physical activity and sleep. This evaluation of engagement with the telephone support, as part of the broader process evaluation of the CHAT program, has shown that there were unintended personal benefits that participants gained from the program. Despite the efforts of intervention providers to contact participants at a time nominated by participants that was convenient to them, intervention providers were unable to reach participants and sometimes up to 10 telephone calls were made before a successful attempt to reach a participant (24). To increase engagement, the program provided participants with the opportunity to nominate their preferred times to receive telephone calls.

Participant engagement has been investigated in child obesity prevention or treatment programs that were community-based (7, 8) or delivered digitally or on-line (17, 18), *via* the internet (19); *via* mobile phone app and email (17) or *via* email, telephone or text messages (27). Engagement with child obesity prevention interventions has been facilitated when the frequency of and access to intervention delivery was more intense *via* the web (19, 32), and a combination of app and email were employed for intervention delivery enabling regular push notifications for intervention exposure (17). Development of programs delivered remotely and flexibly *via* mobile or web has the potential to minimise participant burden and increase program scalability (3). A web-based behaviour change program delivered to adults for healthy body weight and lifestyle established a strong dose-response relationship between the number and intensity of counselling sessions and behaviour change outcomes (19).

### 4.1 Future Directions

Service engagement and program utilisation involves a degree of initiative on the part of parents as well as attempts by service providers to engage with them (9). With regards to successful approaches to engaging parents, the existing research base is far from robust. Parents want services that are reliable, accessible, sensitive to individual needs and well-co-ordinated (9). More research needs to be done on parents’ own help-seeking behaviours and attitudes (9, 37). Despite current availability of mainstream services for psychosocial support, this research has highlighted the need for additional support to meet the psychosocial needs of mothers with infants, for programs such as the CHAT to achieve the intended outcomes. It is important to recognise this for future programs or for scale up of the CHAT program.

### 4.2 Limitations

The main limitation of this study is that we have used secondary data to measure engagement of participants’ engagement with the CHAT program. However, to overcome this limitation we have incorporated analysis of notes taken by intervention providers at the time of intervention delivery. To our knowledge, there is scant published literature on engagement with interventions delivered for obesity prevention practices to draw comparisons, we have attempted to draw comparisons with the limited literature available. Families with scarce resources and without access to telephone were not provided with an opportunity to engage in this program. Providing participants with a range of options to access the program could be considered in future programs.

## 5 Conclusions

Participants’ engagement with the telephone support provided in the CHAT program was good. Engagement with the program interventions and the anticipated behaviour changes were positively associated with certain socio-demographic characteristics. Australian born participants, and participants from a higher socio-economic background associated with household income and employment engaged significantly more with the telephone support provided in the CHAT program. Additionally, the program engaged more participants older than 30 years of age and those who spoke English at home. The program provided unintended personal benefits to some participants with high engagement level due to their various psychosocial needs such as domestic violence, mental health, sleep related issues, which was revealed as part of this evaluation of engagement with the telephone support of the CHAT program. There is limited research on participant engagement and on how to maintain engagement especially in behaviour change programs delivered for infant obesity prevention.

## Data Availability Statement

The original contributions presented in the study are included in the article. Further inquiries can be directed to the corresponding author.

## Ethics Statement

The studies involving human participants were reviewed and approved by Sydney Local Health District Ethics Committee (approval number X16-0360). The patients/participants provided their written informed consent to participate in this study.

## Author Contributions

LW, CR, and LB conceived and designed the original Healthy Beginnings and CHAT RCT project. ME and HX conducted the data analysis with input from LW and CR. ME received doctoral supervision from CR, LB, LW, SM, and ST. ME drafted the manuscript and all authors critically edited and approved the final version.

## Funding

The CHAT RCT is a partnership project funded by the New South Wales Health Translational Research Grant Scheme 2016(TRGS 200) and Sydney Local Health District (SLHD). ME is a PhD scholar funded by the University of Sydney Postgraduate Award scheme.

## Conflict of Interest

The authors declare that the research was conducted in the absence of any commercial or financial relationships that could be construed as a potential conflict of interest.

## Publisher’s Note

All claims expressed in this article are solely those of the authors and do not necessarily represent those of their affiliated organizations, or those of the publisher, the editors and the reviewers. Any product that may be evaluated in this article, or claim that may be made by its manufacturer, is not guaranteed or endorsed by the publisher.

## Abbreviations

CHAT, Communicating Healthy Beginnings Advice by Telephone; RCT, randomised controlled trial; NSW, New South Wales; LHD, Local Health District; AOR, Adjusted Odds Ratio; CI, Confidence Interval; REDCap, Research Electronic Data Capture.
